# Cost-effectiveness analysis of two integrated early childhood development programs into Bangladeshi primary health-care services

**DOI:** 10.1016/j.lansea.2025.100564

**Published:** 2025-03-28

**Authors:** Sheikh Jamal Hossain, Tom Palmer, S.M. Mulk Uddin Tipu, Syeda Fardina Mehrin, Shamima Shiraji, Mohammed Imrul Hasan, Mohammad Saiful Alam Bhuiyan, Nur-E Salveen, Fahmida Tofail, Helen Baker-Henningham, Hassan Haghparast-Bidgoli, Jena D. Hamadani

**Affiliations:** a International Cenre for Diarrhoeal Disease Research Bangladesh (icddr,b), Mohakhali, Dhaka, 1212, Bangladesh; b Global Health and Migration Unit, Department of Women's and Children's Health, Uppsala University, Sweden; c University College London, Institute for Global Health, London, United Kingdom; d Population Health and Immunity Division, Walter and Eliza Hall Institute of Medical Research, Parkville, VIC, Australia, 3052; e School of Psychology and Sport Science, Bangor University, United Kingdom

**Keywords:** Early childhood development, Cost-effectiveness analysis, Parenting, Bangladesh

## Abstract

**Background:**

This study presents results of a cost and cost-effectiveness analysis of two parenting interventions (group-based and pairs) integrated into primary health care centers in rural Bangladesh.

**Methods:**

A within-trial cost-effectiveness analysis was conducted for two trials of parenting interventions aiming to support child development through play and interactions. Eligible participants for both trials were underweight children aged 5–24 months. Participants in the control arms in both trials received standard health services. Intervention costs were estimated rom the provider perspective over the time horizon of each study (21 months for the group-based intervention; 24 months for the pair-based intervention). Incremental cost effectiveness ratios were estimated for all primary child development outcomes and presented in terms of cost per standard deviation improvements in the outcomes. A series of cost scenario analyses were conducted to assess the effect of changing cost assumptions on the cost and cost-effectiveness results. All results are presented in 2022 USD. The studies were registered with ClinicalTrials.gov (NCT02208531).

**Findings:**

Total provider costs in the within-trial analysis were US$ 67,668 for the group-based intervention and US$ 117,028 for the pair intervention. Estimated cost per child covered by the interventions was US$ 156 for the group intervention and US$ 136 for the pair intervention, reflecting likely economies of scale in delivery of the pair intervention. An additional US$ 100 expenditure on the group intervention is estimated to lead to a 0.55 SD improvement in cognition, 0.44 SD in language development and 0.33 SD in motor development. For the pair intervention, the corresponding estimates are improvements of 0.95 SD, 0.81 SD, and 0.88 SD, respectively. Under potential scale up scenarios, the economic cost per child could reduce substantially to US$ 61 and US$ 77 for group and pair interventions, respectively.

**Interpretation:**

The findings indicates that cost-efficiency and cost-effectiveness results for both interventions are comparable with the results from limited similar interventions in LMICs. However, implementation costs of the interventions will be substantially lower at scale due to lower monitoring costs, economies of scale, and full integration into the public health system.

**Funding:**

This work was supported by 10.13039/501100004828Grand Challenges Canada. 10.13039/501100009054ICDDR,B and core unrestricted support form the Government of Bangladesh and the 10.13039/501100000023Government of Canada.

**Research in context**

**Evidence before this study**

Two previous trials of early childhood development (ECD) interventions in rural Bangladesh support the effectiveness of group-based and pair-based interventions in improving child outcomes. However, economic evaluation was not conducted at that time. Additionally, a literature search was conducted in PubMed to identify within trial-economic evaluations of early childhood development interventions, using the terms “economic evaluation” AND “early childhood development” AND “trial”. The search was restricted to publications between Jan 1, 2000 and Nov 10, 2023. Additional citation searching was conducted to further identify relevant studies. Studies taking place in a high-income country, or that only looked at home-visiting interventions, were excluded. Six relevant primary studies were identified: two in China and one in each of India, Kenya, Pakistan and Vietnam. Despite differences in methodologies, all studies broadly conclude that the intervention was cost-effective. However, an additional scoping review identified that compares cost-effectiveness estimates across settings highlights substantial variation in both costs and outcomes.

**Added value of this study**

This study supplements existing evidence on the effectiveness of two interventions with economic analysis, and contributes to scarce evidence on ECD interventions, in particular, those integrated into public healthcare system. Our costs and effectiveness estimates are based on results from two cluster-randomised trials.

**Implications of all the available evidence**

Our analysis showed that the cost-effectiveness of both group-based and pair interventions is comparable with the results from limited similar interventions in LMICs, and that the interventions are potentially scalable in Bangladesh. The implementation costs of the interventions will decrease significantly at scale due to economies of scale, lower monitoring costs and full integration into the primary healthcare system of Bangladesh.

## Introduction

The period from pregnancy to age three is very sensitive for brain development.^1^ Health, wellbeing and learning during this period are the foundation for the future lifecourse.^2^^,^^3^ Accordingly, this is the period when children are most susceptible to environmental influences,^4^^,^^5^ including adversities and risk factors such as extreme poverty, malnutrition, inadequate home stimulation, maternal mental health and insecurity.^6^ Based on proxy indicators of poverty and stunting, 250 million children under five years old are at risk of not reaching their full developmental potential in low and middle income countries.^2^ Poor early development is predictive of lower educational attainment and subsequent lower adult income.^7^^,^^8^ Indeed, deficiencies in early age are compounded and become gradually more difficult to repeal beyond early childhood.^9^

The Global Strategy for Women's, Children's and Adolescents' Health 2016–2030 synthesized a new vision under the objectives of Survive, Thrive and Transform.^10^ Accordingly, the World Health Organization (WHO), World Bank Group, United Nation Children Fund (UNICEF) and United Nation Economic and Social Cooperation (UNESCO) are prioritising programs delivered in early childhood.^11^ In Bangladesh, although the government established the “Comprehensive Policy on Early Childhood Care and Development” in 2013 targeting children aged 0–8, there remains a lack of ECD interventions at scale for children aged 0–3 years. Data to understand ECD status for children aged 0–3 are also limited, while modelled estimates suggest that only 32% of children aged 3–4 years in Bangladesh receive minimally adequate nurturing care.^12^

A number of randomized controlled trials of psychosocial stimulation interventions conducted in low- and middle-income countries (LMICs), including Bangladesh, China, India, Pakistan, Brazil, Colombia documented that psychosocial stimulation can improve children's cognitive, language and motor development and their behaviour.^13^, ^14^, ^15^, ^16^, ^17^, ^18^, ^19^, ^20^ Many of these interventions used the primary health care system,^16^^,^^21^^,^^22^ while others used conditional^18^ and unconditional cash transfer platforms,^23^ or home visits targeting specific populations, such as maltreated children.^24^ Some of the interventions showed sustained benefits in middle childhood and adulthood.^25^, ^26^, ^27^ Investment in early childhood can therefore potentially benefit individuals across the life course and help reduce social inequalities.^28^^,^^29^ Most of these trials were implemented at small scale and were intensive, which may limit scalability and increase costs. The importance of low cost and scalable interventions that are integrated within healthcare systems has been emphasized by the Lancet series on ECD and other ECD researchers and policy makers.^30^ However, effectiveness and cost-effectiveness evidence for these interventions are scarce, particularly in low- and middle-income countries.^31^ This dearth of evidence represents a key obstacle to their large scale implementation.^32^

To fill this gap, two parallel cluster randomized controlled trials were designed and implemented in rural Bangladesh to test the effectiveness of two parenting interventions integrated into Bangladeshi primary health-care services.^21^^,^^22^ The trials evaluated the impact of fortnightly group-based parenting sessions (hereinafter referred to as ‘group-based’ intervention)^22^ or fortnightly pairs of mother-child dyads sessions (hereinafter referred to as ‘pair’ intervention),^21^ both facilitated by government health workers. Both interventions significantly improved children's cognition, language, and motor development and child behaviour, but had no significant effect on children's growth.^21^^,^^22^

This study presents detailed results from the cost and cost-effectiveness analysis of both interventions, along with findings on their cost and affordability at scale.

## Methods

### Study design

The studies were RCTs within the primary health care system of Bangladesh. Detailed descriptions of both group-based and pair trials and the interventions are presented elsewhere.^21^^,^^22^ In brief, the *group-based parenting* was conducted in 40 community clinics (CC) in the Kishorganj district of Bangladesh. The *mother-child pair* trial was implemented in 90 CCs in Narsingdi district. Both studies were conducted as two-arm and single blind. In these trials, the selected CCs or clusters (40 in group-based and 90 in pair) were stratified by subdistrict and then randomly allocated to the intervention or control arms. The clinic was chosen as the unit for randomization to minimize the risk of contamination between the arms. The intervention was integrated into clinic services and carried out by the existing clinic staff.

Written informed consent was obtained from mothers at enrollment. Ethical approval was granted by the institutional review board of the International Centre for Diarrhoeal Diseases Research, Bangladesh (icddr,b).

### Study setting and participants

Kishorgonj and Narshingdi, two adjacent disctricts, were selected to conduct evaluations of group-based and pair-based intervention. Two rural sub-districts were selected for the group-based intervention in Kishorganj District, located approximately 100 km from Dhaka, the capital city of Bangladesh (N = 434). The pair trial was implemented in three rural sub-districts in Narsingdi district, Bangladesh. The district is in the centre of the country, 50 km north-east of Dhaka (N = 859). Both interventions were delivered from CCs by front-line health workers. Each CC was designed to provide primary health care services through a trained Community Health Care Provider (CHCP) for a population of at least 6000. Health Assistants (HA) and Family Welfare Assistants (FWA) also provide health services from the CC alternatively in every three days of a week in their assigned CC. CHCPs and HAs typically hold a bachelor's or master's degree, while FWAs have typically completed higher secondary education. CCs offer general illness management e.g. diarrhea, family planning advice and provide logistics, provide care for pregnant women (including iron and folate supplementation), health, hygiene and nutrition education, monitor child growth, and make referrals to other healthcare facilities. The primary health care system in both districts is comparable to other districts in Bangladesh.

Eligible participants for both trials were underweight children aged 5–24 months and their mothers/caregivers who were living within 30 min walking distance from the households to CCs. However, the sample in each CC was restricted to a maximum of 25 children, for pragmatic reasons. Underweight was defined as a weight-for-age Z score of −2 SDs of the WHO standard^33^ for the pair intervention, and −1.5 SDs of the WHO standard for the group intervention.

### Description of the interventions and comparators

#### Interventions

Both interventions consisted of 25 fortnightly sessions, in which mothers/caregivers were shown how to support their child's development through play and interactions. In addition, mothers/caregivers were lent books and toys to take home after each session. The primary difference between the interventions was the number of mother-child dyads in the sessions. In the group-based intervention, an average of four mother-child dyads in each group attended the sessions, and the group size was limited by the available space within the clinic. While in the pair intervention, a pair of mother/child dyads (two mothers and their children) attended the sessions. The mean number of mother-child dyads per clinic was 21 for the group intervention (maximum 24) and 20 for the pair intervention (maximum 25). Participants were not provided with incentives, though children did receive biscuits during the sessions.

We developed the intervention sessions based on the Reach-Up and Learn curriculum, which was adapted from the Jamaican Reach-Up home visiting program.^34^ The play sessions with mothers and children were participatory and adhered to a predetermined structure. Each session included a review of previous home activities with discussion, a local song, engaging activities with a picture book and developmentally appropriate toys, language-based activities, the delivery of nutritional messages, and a review of tasks to be continued at home. Each session had specific activities and materials tailored to the child's developmental stage, ensuring that they were both stimulating and suitable for the child's abilities. However, the curriculum used for the pair intervention targeted activities according to the child's age in months, while the group intervention activities were divided into broader age bands (e.g. 12–18 months).

Health workers, including CHCPs, FWAs, and HAs, who were stationed at each community clinic, administered the play sessions, which typically lasted 40–60 min (40–50 min for the group-based intervention). Prior to the initiation of the intervention, these health workers underwent a comprehensive 10-day training on the curriculum. Additionally, they received a half-day refresher training every three months. The supervisors of these health workers also underwent a one-day training session to enhance their understanding of the intervention. CHCPs and HAs were overseen by Assistant Health Inspectors (AHIs), while Family Planning Inspectors (FPIs) supervised the FWAs. Finally, project staff provided additional supervision to the health workers to maintain the quality and effectiveness of the intervention and to ensure proper monitoring. Supervisors with a master's degree in psychology or another related subject were recruited and trained. Each supervisor monitored four CCs, meeting each health worker twice per month.

#### Control arm

Participants in the control arms in both trials received standard services provided by the health workers at the clinics. More detail on each intervention and study design is provided in published impact analyses.^21^^,^^22^ It is worth noting that both interventions were evaluated with respect to separate control arms only, and were not directly compared.

#### Outcomes/effectiveness

Outcomes in both trials were measured in the comparison and intervention group clinics, at baseline and after 1 year of the interventions. In the group intervention, all participants were evaluated.^22^ In the pair intervention, a randomly selected subsample of eight mother–child pairs from each of the intervention and control community clinics formed the evaluation sample, amounting to around 40% of participants were evaluated.^21^ This evaluation sample size was based on power calculations for analysis of the primary outcome.

##### Primary outcomes

The primary outcomes were children's development and their behaviour and growth. We measured children's cognitive, language and motor development using the Bayley Scales of Infant and Toddler Development (3rd version).^35^ We measured behavior scores based on Wolke's behaviour rating scale.^36^ Child behavior was rated using four scales: approach to examiner, emotionaltone, cooperativeness, and vocalizations. Trained testers also measured children weight and length/height using WHO standard methods.^37^ Z-scores of weight-for-age, weight-for-height, and height-for-age were calculated using WHO AnthroPlus.

##### Secondary outcomes

The secondary outcomes were maternal knowledge on child care, quality of home stimulation and mothers’ depressive symptoms. Maternal knowledge was measured using a specially designed instrument consisting of 20 questions.^23^ We measured quality of home stimulation using Family Care Indicator, a validated tool in this context.^38^ We also collected maternal depressive symptoms using six questions taken from the Center for Epidemiological Studies Depression Scale.^39^

Statistical analyses accounted for clustering and were adjusted for baseline characteristics. More details on the analysis, outcomes and measurement tools are presented elsewhere.^21^^,^^22^

#### Cost analysis

Base case analysis was conducted as a within-trial analysis, accounting for the full economic cost of implementing the intervention. Economic costs of each intervention were estimated from the provider perspective, including costs incurred by the implementing partner (icddr,b; program costs) and costs incurred by the Bangladesh Ministry of Health and Family Welfare (provider costs). Total costs include both start-up costs, such as training, and implementation costs of the intervention, including facility staff and materials. Cost data were collected by field research assistants with Master's degrees in health economics, who liaised with government employees (health care providers) and project personnel.

All programme costs were assessed over the full time horizon of each study. The time horizon for the pair-based study was January 2014 to December 2015 (24 months), and February 2015 to October 2016 for the group-based intervention (21 months). Both interventions had an initial start-up period of 9 months for preparation activities and to develop intervention materials. This was followed by the implementation period, which was 12 months for the group-based intervention. Roll-out of the pair-based intervention was staggered across CCs, resulting in a total implementation period of 16 months. However, the duration of the intervention was 12 months for each individual participant in both studies.

A combination of activity-based, expenditure and ingredient approaches were used to estimate costs.^40^ All cost analysis was conducted retrospectively. Program costs were based on analysis of project accounts. All included program costs are intended to reflect the costs of intervention design, monitoring and evaluation (M&E) and quality assurance only, excluding research costs. This allocation of costs was based primarily on time-use survey data for program staff, which was used to disaggregate total staff salary costs. Where time allocation data were unavailable, estimates were assumed through consultation with the principal investigator and project coordinator. Additionally, travel costs were allocated to the intervention (i.e., excluding research costs) based on interviews with relevant project staff and information from program financial reports. As a detailed disaggregation of other costs incurred by the implementing partner was not possible, we conservatively assumed that the proportion of all other implementing partner costs (including overheads and consumable costs) that should be allocated to the intervention reflect the staff time-use allocation to the intervention.

Recurrent costs including intervention materials, toys, books and refreshments were directly allocated to the interventions, as were start-up costs related to training. Structured questionnaires were used to collect provider cost data from CCs, using a sample of five randomly selected CCs delivering the pair intervention, and three randomly selected CCs delivering the group intervention. For each intervention, average costs per child were estimated by dividing total costs across the sampled facilities by the number of children receiving the intervention in those facilities. This average cost per child was then used as the basis for estimates across all intervention facilities. The total estimated facility costs include the costs of staff directly involved in the intervention, facility overhead costs attributable to the intervention, and capital costs. Capital items were annualized for their respective functional lifetime.

To help guide local policymakers, two alternative cost at scale scenarios were explored in the analysis. First, cost at scale scenario 1 assumes that only 10% of within-trial program costs would be incurred in the event of intervention scale-up, while facility costs would remain the same. This reflects that program costs (i.e., M&E, quality assurance and intervention design) comprise a disproportionately high proportion of costs in the within-trial analysis, but would likely not be incurred if the intervention were to be scaled up domestically. However, some degree of quality assurance would still be required at scale to ensure effectiveness is maintained. All facility implementation costs are included in this scenario. Second, cost scenario 2 excludes both program costs and facility costs, and reflects the costs of training, intervention materials, and consumables only. This reflects that adoption of the intervention may involve a reallocation of existing resources, rather than requiring new investment in staff and facilities. This assumes that there is existing capacity to adopt the intervention, and does not capture opportunity costs. Thus, cost scenario 2 broadly reflects the financial cost of intervention implementation, and is presented as an exploratory analysis.

Costs were estimated in Bangladeshi Taka (BDT) and inflated to 2022 base year values using local inflation rates.^41^ These costs were then converted to USD (exchange rate of 91.7).^42^ Base-case estimates assume an annual discount rate of 3% to convert to present values. All costing and analysis was conducted in Microsoft Excel. Estimated costs per child provide a measure of cost-efficiency, which may also be useful to inform resource allocation.

#### Cost-effectiveness analysis

Incremental cost-effectiveness ratios (ICERs) were calculated by dividing incremental costs by incremental outcomes of the interventions. ICERs were estimated for all primary child development outcomes for which an effect of the intervention was detected. For the purposes of the cost-effectiveness analysis, all outcomes are presented in terms of standard deviation improvements in outcomes per currency unit.

A series of cost scenario analyses were conducted to assess the effect of changing cost assumptions on the cost and cost-effectiveness results. Base case ICERs are compared with ICERs generated based on both cost scenario 1 and 2 (see above). Further one-way sensitivity analyses were also conducted to explore the impact of uncertainty in other parameters on results. Intervention costs were varied by ±25%, trial outcomes were varied based on their 95% confidence intervals, and alternative discount rates of 0% and 6% were used.

#### Cost and affordability at scale

The potential cost of delivering the interventions at scale was estimated. A maximum capacity of 416,000 children a year was assumed, based on previous estimates of the capacity of the 13,000 excisting community clinics nationally in Bangladesh.^22^ While we do not explicitly address targeting criteria in this article, targeting could be based on weight criteria, particularly given our effectiveness estimates are for underweight children. The total cost of this expansion was estimated based on both cost scenario 1 and 2, and compared with the annual budget of the Bangladesh Ministry of Health and Family Welfare^43^

#### Role of the funding source

This work was supported by Grand Challenges Canada. icddr,b also reports core unrestricted support form the Government of Bangladesh and the Government of Canada. The funder had no role in the design of the study, data collection, data analysis, data interpretation, or writing the report.

## Results

### Provider cost analysis

Table 1 summarises estimated within-trial provider costs for both interventions. Total provider costs were US$ 67,668 for the group intervention and US$ 117,028 for the pair intervention. Program costs account for a majority of costs for both the group intervention (68%) and pair intervention (48%). The total estimated cost per child is US$ 156 for the group intervention and US$ 136 for the pair intervention in the within-trial analysis. Although this may appear counterintuitive as the group-based intervention is in theory less intensive, this reflects the large proportion of program costs, where there may be economies of scale in monitoring activities, for examples the number of participants in the pair intervention was more than double that in the group intervention. Although program costs are higher for the pair intervention than for the group intervention in the within-trial analysis, the per child costs are lower, reflecting that some minimum level of program costs are likely required to establish an intervention. Alternative cost assumptions at scale are explored below.

**Table 1:** Within-trial program costs for total intervention period.

	Group cost, US$ (n = 434)	Pair cost, US$ (n = 859)	Description
Facility staff	2570	16,816	Including salary cost of facility staff for the time spent delivering the intervention.
Facility capital	173	1177	Costs of capital items, including furniture, were annualized for their respective functional lifetime.
Facility overheads	189	1960	Including facility rental costs.
*Subtotal facility costs*	2932	19,953	
Motivational workshops	7033	13,654	Before the start of the intervention, community motivational meetings were held in each area to encourage participation, and every 4 months, a refresher meeting was held in each village for all participating mothers and other family members to sustain engagement in the program. We ran three monthly workshops for the staff, which accrued a cost that included transport and refreshments, but these workshops appeared necessary to keep staff motivated and solve problems.
Training	4701	8786	Ten days of initial training for health workers delivering interventions, including stipends, materials and refreshments (N = 130 pair-based, N = 58 group-based).
*Subtotal workshops and training*	11,734	22,440	
Toy making staff	1579	4291	Payments to staff who made toys for play sessions.
Intervention materials and consumables	5656	13,608	Including toys, printed materials, refreshments for intervention sessions
*Subtotal materials and consumable costs*	7235	17,899	
Program staff	30,655	37,601	Salary costs of staff time for intervention design, monitoring and evaluation, and quality assurance.
Program travel	5436	7462	Cost of travel to intervention clinics.
Other program costs	9674	11,674	Including overheads and office supplies.
*Subtotal program costs*	45,765	56,737	
Total provider costs	67,668	117,028	
Total provider costs per child	156	136	

### Intervention outcomes

Table 2 displays outcomes from the trial impact analysis for each intervention. Primary outcome measures included child development assessed using the Bayley scale. The group-based intervention was found to have significant benefits of intervention to child cognition (effect size 0.85 SDs, 95% CI: 0.59, 1.11), language (0.69 SDs, 0.43, 0.94), and motor development (0.52 SDs, 0.31, 0.73). The pair intervention also significantly improved children's cognition (1.3 SDs, 1.1, 1.5), language (1.1 SDs, 0.9, 1.2), and motor development (1.2 SDs, 1.0, 1.3).

**Table 2:** Trial child development outcomes.

Trial outcomes	Group intervention effect size, SDs [95% CI]	Pair intervention effect size, SDs [95% CI]
Cognition	0.85 [0.59–1.11]	1.3 [1.1–1.5]
Language	0.69 [0.43–0.94]	1.1 [0.9–1.2]
Motor	0.52 [0.31–0.73]	1.2 [1.0–1.3]

### Within-trial cost-effectiveness analysis

Table 3 reports base case ICERs for each intervention, and for each of the outcomes. Higher ratios imply a larger impact on child development per given amount of expenditure. As estimated intervention effect sizes are larger for the pair intervention, and the estimated cost per child is lower, the pair intervention is more cost-effective in the base case analysis. In the base case provider perspective analysis, an additional US$ 100 expenditure on the group intervention is estimated to lead to a 0.55 SD improvement cognition, 0.44 SD in Language development and 0.33 SD in motor development. For the pair intervention, the corresponding estimates are improvements of 0.95 SD, 0.81 SD, and 0.88 SD respectively.

**Table 3:** Within-trial cost-effectiveness analysis results.

Incremental cost-effectiveness ratios (Additional SD per US$ 100)	Group intervention	Pair intervention
Cognition	0.55	0.95
Language	0.44	0.81
Motor	0.33	0.88

### Cost at scale scenario analyses

In cost scenario 1, excluding 90% of program costs from the cost estimates results in an estimated cost of US$ 61 per child for the group intervention and US$ 77 for the pair intervention (Table 4). Further excluding facility costs (in addition to excluding the remaining 10% of program costs) in cost scenario 2 has a relatively small influence on the less labour intensive group intervention, reducing costs to US$ 44 per child, while a larger reduction in costs is observed for the pair intervention, which would cost US$ 47 per child under this assumption. As all of these costs are less than US$ 100 per child, cost-effectiveness ratios for these cost scenarios were not calculated.

**Table 4:** Cost at scale scenario analyses.

	Group intervention	Pair intervention
**Base case analysis**		
Total cost	67,529	117,726
Cost per child	156	136
**Cost at scale scenario 1**^a^		
Total cost	26,340	66,662
Cost per child	61	77
**Cost at scale scenario 2**^b^		
Total cost	18,831	40,478
Cost per child	44	47

a Including 10% of within-trial program costs, and 100% of within-trial facility costs.

b Including costs of training, intervention materials, and consumables only.

### Other sensitivity analyses

Appendix 1 shows the impact of changes in discount rates and trial outcomes on the results of the cost-effectiveness analyses. Discount rates had limited influence on results. Variation in costs and outcomes has greater influence on results, though all ICERs for both interventions remained at a minimum of 0.2 additional SD per US $100.

### Cost and affordability of scale up

Scaling up the intervention to 416,000 children annually in Bangladesh would cost around US $25.4 million for the group intervention and US$ 31.9 million for the pair intervention under cost scenario 1. This amounts to around 0.71% and 0.90% of the annual national health budget respectively. Under cost scenario 2, where facility costs are removed, the equivalent figures are 0.51% and 0.55% respectively. It is likely that further economies of scale in expenditure categories such as intervention materials could reduce this cost in the event of scale up.

## Discussion

We conducted an economic evaluation of two parenting interventions integrated into primary health care centers in rural Bangladesh. Estimated total provider costs per child were US$ 156 for a group-based intervention and US$ 136 for a pair intervention. The group-based intervention was more expensive in the within-trial analysis, given higher program costs (i.e., M&E and quality assurance), and had a smaller impact on child outcomes when compared with the pair intervention. Although at scale the group-based intervention would be cheaper, and be less resource-intensive within facilities, the trial was much smaller than that of the pair-based intervention, with around half the number of participants, and therefore per child program costs were higher. In the within-trial analysis, the group intervention had effect sizes per US$ 100 expenditure of 0.55 SD in cognition, 0.44 SD in language and 0.33 in motor, while the equivalent effect sizes for the pair-based intervention were 0.95, 0.81 and 0.88.

This study contributes to limited global evidence on the cost and cost-effectiveness of parenting interventions. Relevant studies of comparable group interventions include a study of a group intervention in India which estimated that group sessions cost $38 per child per year.^44^ In Kenya, an evaluation of a group-based parenting intervention estimated provider costs of US$ 119 per child.^45^ Finally, in Vietnam, a study estimated total provider costs for a group intervention of US$ 234 per child.^46^ Comparison across studies is hindered by differences in intervention group size, intensity, duration, and context. However, none of the interventions referred to above were integrated within health systems, which may facilitate expansion to larger scale implementation. In China, two interventions that were integrated within existing primary health services cost US$ 50.87^47^ and US$ 146.10 per child,^20^ though again differences in costing methodologies limit comparisons. Additionally, the two interventions in Bangladesh differ from these other studies, as only underweight children in rural areas were eligible. The effect sizes for the interventions in the present study are also comparatively large. For example, in comparison, effect sizes in Kenya were 0.52 SD for cognition and 0.42 SD for receptive language, while in India they were 0.28 SD for cognition and 0.30 for language. This compares to respective effect sizes of 0.85 SD and 0.69 SD for the group-based intervention and 1.3 SD and 1.1 SD for the pair intervention.

Verguet et al.^45^ propose a framework to improve comparability of cost-effectiveness results across interventions by dividing incremental costs by either the average or the sum of domain-specific effects on child development outcomes.^48^ Using the former approach without standardizing costs results in ICERs of US$ 227.06 and US$ 113.53 per 1 SD improvement for the group-based and pair interventions respectively. For the latter approach the estimates are US$ 75.69 per 1 SD and US$ 37.84 per 1 SD. If these two interventions were added to the 12 analysed by the authors, they would be the 8th and 5th most cost-effective when using average effect sizes, or the 6th and 5th most cost-effective when using summed effect sizes. Of course, such comparisons should be treated with caution given differences in the measurement of both costs and outcomes. Additionally, ICERs expressed per 1 SD improvement in outcomes must also be interpreted with caution, as interpretation depends on the size of the SD, and given that most interventions do not reach effect sizes of this magnitude. For example, one study claims that “the intervention delivers a 1 SD improvement in infant cognitive development for $4.56”, despite only estimating a 0.057 SD improvement on an observed child development index.^49^ However, despite these limitations, such comparisons may provide useful indicative evidence that the cost-effectiveness of group-based and pair interventions is broadly in-line with other relevant studies.

Implementation costs of the two interventions may also be substantially lower at scale due to lower monitoring costs, economies of scale and full integration into the public health system. Indeed, both the studies were monitored by project staff mainly, leading to high program costs. In alternative cost scenarios where program costs are excluded, the pair intervention (US$ 77 per child) is more expensive than the group intervention (US$ 61 per child). Similarly, when both program and facility costs are excluded, the pair intervention cost US$ 47 per child, compared with US$ 44 per child for the group intervention. However, in the event of scale-up, it would be important to closely monitor whether implementation quality is maintained, as supervision and training responsibilities would fall upon government rather than program staff. Finally, alternative cost scenarios explored in this analysis assume capacity to absorb the intervention without substantial reallocation from other valuable activities, which may not be realistic. Additionally, the targeting of the intervention to risk groups during this scale-up was not explicitly considered in the analysis. However, under current capacity and group sizes, it would only be possible to reach around 14% of the approximately 3 million children born each year in Bangladesh, meaning some targeting would be necessary, such as targeting underweight children. All decisions regarding program scale up in a given context should account for multiple considerations beyond cost-effectiveness,^50^ including intervention acceptability and feasibility.

This analysis has several unavoidable limitations. First, the analysis was retrospective and was conducted several years after the intervention was implemented, meaning that some institutional knowledge regarding certain costs was lost over time, and several assumptions were required to allocate program staff time-use to interventions. Second, despite comparatively large impacts observed in the short term, it is unclear whether these can be sustained in the long term, and how they translate to future outcomes. There is a lack of follow-up studies of ECD interventions to evaluate whether observed benefits are sustained, and this should be a future research priority. Third, it was not possible to standardize outcomes, and therefore, as in other related studies, ICERs are presented separately for different domains of child development. Fourth, the impact of both the group- and pair-based interventions was assessed relative to separate control groups only, and it is not possible to directly compare the interventions, which differed in terms of study size, location and weight-for-age of participating children. Comparisons of relative cost-effectiveness between the two interventions, and similar interventions elsewhere, should therefore be treated with caution. In particular, participating children were underweight, a key risk factor for poor early childhood development, and therefore intervention effect sizes may not generalizable to the overall child population. Finally, available data enabled a provider perspective cost-effectiveness analysis. A societal perspective would capture the costs and benefits of the intervention more comprehensively, and the intervention costs would increase through capturing the opportunity cost of household participation. A full cost-benefit analysis would further capture future benefits, such as the impact of improved cognitive development on future earnings, for example. However, it remains uncertain whether the benefits of ECD interventions can be sustained long-term, and further evidence is needed.

### Conclusion

The findings indicates that cost-efficiency and cost-effectiveness results for both interventions are comparable with the results from limited similar interventions in LMICs. However, implementation costs of the interventions will be substantially lower at scale due to lower monitoring costs, economies of scale, and full integration into the public health system.

## Contributors

Sheikh Jamal Hossain: Conceptualisation, data curation, formal analysis, investigation, methodology, writing-original draft.

Tom Palmer: Formal analysis, methodology, writing–review & editing.

S.M. Mulk Uddin Tipu.

Syeda Fardina Mehrin, Shamima Shiraji, Mohammed Imrul Hasan, Mohammad Saiful Alam Bhuiyan.

Nur-E-Salveen.

Fahmida Tofail.

Helen Baker-Henningham: Conceptualisation, funding acquisition, investigation, writing–reviewing and editing.

Hassan Haghparast-Bidgoli: Conceptualisation, methodology, formal analysis, supervision, writing–reviewing and editing.

Jena D Hamadani: Conceptualisation, funding acquisition, investigation, supervision, writing–reviewing and editing.

## Data sharing statement

Data available on request with the corresponding author.

## Declaration of interests

We declare no competing interests.
