# Supplementary material for: Cost-effectiveness analysis of two integrated early childhood development programs into Bangladeshi primary health-care services
Source: Lancet Reg Health Southeast Asia. 2025 Mar 28;35:100564. doi: 10.1016/j.lansea.2025.100564 (PMC11995800; doi:10.1016/j.lansea.2025.100564)
Supplement: Appendix [file mmc1.docx]

**Appendix 1 – Deterministic one-way sensitivity analysis results**

| **Parameter** | **Group intervention**  **(Additional SD per US$100)** | | | **Pair intervention**  **(Additional SD per US$100)** | | |
| --- | --- | --- | --- | --- | --- | --- |
|  | **Base case** | **Minimum** | **Maximum** | **Base case** | **Minimum** | **Maximum** |
| **Discount rate*** | | | | | | |
| Cognition | 0.55 | 0.53 | 0.56 | 0.95 | 0.92 | 0.99 |
| Language | 0.44 | 0.43 | 0.46 | 0.81 | 0.78 | 0.84 |
| Motor | 0.33 | 0.32 | 0.35 | 0.88 | 0.85 | 0.91 |
| **Outcomes**^#^ | | | | | | |
| Cognition | 0.55 | 0.38 | 0.71 | 0.95 | 0.81 | 1.10 |
| Language | 0.44 | 0.28 | 0.60 | 0.81 | 0.66 | 0.88 |
| Motor | 0.33 | 0.20 | 0.47 | 0.88 | 0.73 | 0.95 |
| Intervention costs^ | | | | | | |
| Cognition | 0.55 | 0.44 | 0.73 | 0.95 | 0.76 | 1.27 |
| Language | 0.44 | 0.35 | 0.59 | 0.81 | 0.65 | 1.08 |
| Motor | 0.33 | 0.27 | 0.44 | 0.88 | 0.70 | 1.17 |

**Varied between 0% and 6%; # varied between 95% CIs (see Table 2); ^ varied by +/- 25%.*
